# Association of SGLT2 inhibitor dapagliflozin with risks of acute kidney injury and all-cause mortality in acute myocardial infarction patients

**DOI:** 10.1007/s00228-024-03623-7

**Published:** 2024-02-06

**Authors:** Dabei Cai, Qianwen Chen, Lipeng Mao, Tingting Xiao, Yu Wang, Qingqing Gu, Qingjie Wang, Yuan Ji, Ling Sun

**Affiliations:** 1 Department of Cardiology, the Affiliated Changzhou Second People’s Hospital of Nanjing Medical University, Changzhou, Jiangsu 213000 China; 2 Dalian Medical University, Dalian, Liaoning, 116000 China

**Keywords:** Acute myocardial infarction, Acute kidney injury, Sodium-glucose cotransporter inhibitor, Dapagliflozin, Mortality

## Abstract

**Objective:**

Sodium-glucose cotransporter 2 (SGLT2) inhibitors have well-documented effects in reducing hospitalization or cardiovascular mortality, while the association of SGLT2 inhibitor dapagliflozin (DAPA) and the risk of acute kidney injury (AKI) in acute myocardial infarction (AMI) patients has not been comprehensively investigated. Therefore, we aimed to assess the association between DAPA and AKI risk in AMI patients after percutaneous coronary intervention (PCI) therapy.

**Methods:**

Using the Changzhou Acute Myocardial Infarction Registry database, we retrospectively included AMI patients from January 2017 to August 2021 and analyzed the risk of AKI and all-cause mortality after PCI therapy. The patients were divided into two groups according to the use of DAPA (DAPA group and Ctrl group). Patients in the DAPA group started to use DAPA after admission and continued its use during hospitalization and follow-up period. Baseline characteristics were balanced between the two groups with a propensity score matching (PSM) analysis. The outcome was AKI within 7 days after PCI and all-cause mortality during a follow-up of 2 years. Univariate and multivariate logistic regression analyses were used to assess the association between DAPA and AKI risk.

**Results:**

A total of 1839 AMI patients undergoing PCI were enrolled. DAPA was used in 278 (15.1%) patients. Postoperative AKI occurred in 351 (19.1%) cases. A 1:1 PSM analysis was used to reduce confounding factors. The multivariate stepwise regression analysis showed that DAPA (odds ratio, OR 0.66; 95% confidence interval, CI 0.44–0.97; *P* = 0.036) was an independent protective factor in the entire cohort. After matching, the use of DAPA in AMI patients was independently associated with a decline of AKI risk (OR 0.32; 95% CI, 0.19–0.53; *P* < 0.001) after hospital admission. Meanwhile, there were significant differences in mortality between the DAPA group and Ctrl group (2.5% vs. 7.6%, *P* = 0.012).

**Conclusion:**

SGLT2 inhibitor DAPA was associated with lower risks of incident AKI and all-cause mortality in AMI patients after PCI therapy.

**Supplementary Information:**

The online version contains supplementary material available at 10.1007/s00228-024-03623-7.

## Introduction

Percutaneous coronary intervention (PCI) therapy can be used to treat acute myocardial infarction (AMI), a common emergency with high mortality and morbidity worldwide [1–3]. AMI patients may develop acute kidney injury (AKI) after admission, due to factors such as age, baseline renal function, use of contrast agents, no-reflow phenomena, and complex coronary artery anatomy [4]. James et al. have reported a 9.6% incidence of AKI among 14,782 participants undergoing coronary angiography [5]. AKI is associated with increased in-hospital and long-term mortality [6, 7]. Hence, renal protection strategies before PCI may effectively reduce the risk of contrast-induced acute kidney injury (CI-AKI) and long-term mortality among AMI patients who are undergoing PCI [8].

Dapagliflozin (DAPA) is a sodium-glucose cotransporter 2 (SGLT2) inhibitor approved in China in 2017. Clinical trials have shown that SGLT2 inhibitors improve the cardiovascular and renal outcomes in people with or without diabetes mellitus (DM) [9–12], and it has been approved by the Japanese Circulation Society/Japanese Heart Failure Society (JCS/JHFS) 2021 Guideline Focused Update on Diagnosis and Treatment of Acute and Chronic Heart Failure [12]. Basic studies have also proven that SGLT2 inhibitor empagliflozin attenuates kidney injury after myocardial infarction in DM rats [13]. Yet, whether empagliflozin is beneficial to AMI patients undergoing PCI has not been answered.

In this study, we aimed to evaluate the effects of DAPA which was started after hospital admission on the risk of AKI and all-cause mortality in patients with AMI using propensity score matching (PSM) analysis.

## Materials and methods

### Patients

The clinical data of 1839 AMI patients who received PCI therapy in the Affiliated Changzhou Second People’s Hospital of Nanjing Medical University from January 2017 to August 2021 were retrospectively analyzed. Included were patients (1) diagnosed with AMI; (2) aged 18–90 years; and (3) had received PCI therapy during the hospitalization. The diagnosis of AMI was based on the European Society of Cardiology/American College of Cardiology (ESC/ACC) diagnostic criteria for AMI [14]. The exclusion criteria were (a) end-stage kidney disease (estimated glomerular filtration rate (eGFR) ≤ 15 mL/min, or requiring renal replacement therapy). eGFR was calculated according to the Modification of Diet in Renal Disease (MDRD) equation eGFR(mL/min/1.73 m^2^) = $186{\left({\text{Scr}}\right)}^{-1.154}\times {{\text{age}}}^{-0.203}\times \left(0.742\mathrm{ if female}\right)$ [15]; (b) absence of baseline and (or) postoperative creatinine; (c) use of other kinds of SGLT2 inhibitors during hospitalization and (or) follow-up period; (d) use of DAPA before admission; (e) initiation of DAPA after discharge and the follow-up period; and (f) discontinuation of DAPA during hospitalization or after discharge for various reasons.

Among the enrolled patients, the AMI patients accompanied by type 2 diabetes and (or) heart failure with reduced ejection fraction (HFrEF) were given DAPA treatment. Type 2 diabetes was diagnosed based on WHO criteria [16]: (1) glycosylated hemoglobin (HbA1c) ≥ 6.5%; (2) fasting blood glucose ≥ 7.0 mmol/L; (3) random blood glucose ≥ 11.1 mmol/L with concomitant osmotic symptoms. HFrEF was defined as a complex clinical syndrome characterized by structural and/or functional impairment of the left ventricle, resulting in a decrease in heart pump function (left ventricular ejection fraction (LVEF) ≤ 40%) [17].

**Fig. 1:**
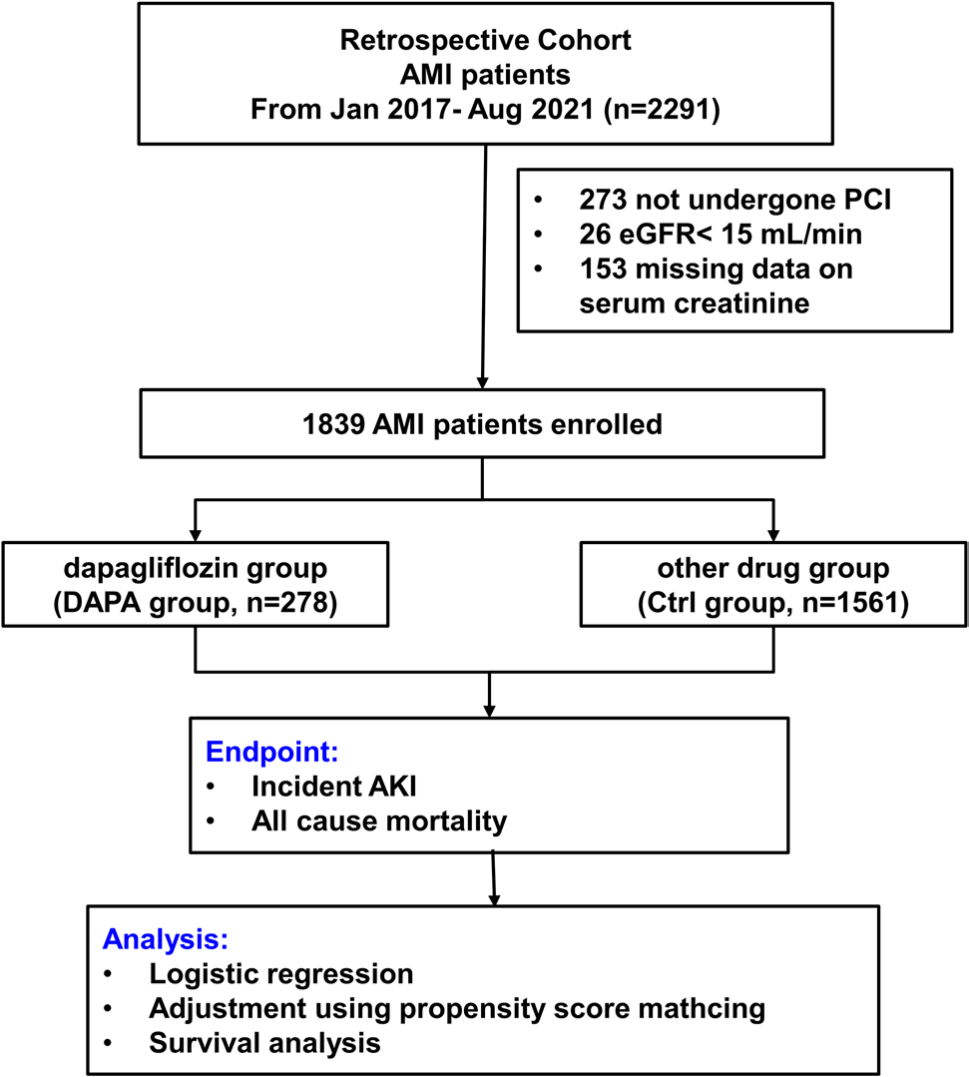
Study flow chart. AMI acute myocardial infarction, PCI percutaneous coronary intervention

The enrolled patients were then divided into two groups: the DAPA group (*n* = 278) and the Ctrl group (*n* = 1561), according to whether DAPA was applied. Patients in the DAPA group started to use DAPA after admission and continued its use during hospitalization and follow-up period. A flow chart of the present study is shown in Fig. 1.

### Ethical consideration

The studies involving human participants were reviewed and approved by the Ethics Committee of the Affiliated Changzhou Second People’s Hospital of Nanjing Medical University and carried out in accordance with the principles of the Declaration of Helsinki. The ethics approval number was 2020-KY253-01. The requirement for informed consent forms for the patients was waived by our institution due to the retrospective nature of the study.

### Data collection

The data in this study were retrieved from the electronic inpatient medical record system of the Affiliated Changzhou Second People’s Hospital of Nanjing Medical University. The clinical data of enrolled patients were collected, including demographic information, medical history, laboratory indicators, and operation-related indexes.

### Study endpoints

The primary outcome was defined as the occurrence of AKI within 7 days after PCI therapy. AKI was confirmed under either of the following conditions: (1) a serum creatinine level increased by more than 0.3 mg/dL within 48 h; or (2) a 1.5-fold increase in serum creatinine from baseline within 7 days [18]. The baseline plasma creatinine was defined as the first creatinine level measured after admission. The secondary endpoint was all-cause mortality in the enrolled cohort. Mortality data of the enrolled patients were obtained by telephone or outpatient interview during the follow-up by investigators. The enrolled patients were followed up for 2 years.

### Statistical analysis

The Kolmogorov–Smirnov test was used to assess the normal distribution of continuous variables. Continuous variables were expressed as mean ± standard deviation (SD) and compared using *t*-tests. If the continuous variables were not normally distributed, they were then expressed as median, 25th and 75th percentile, and compared using a non-parametric test. Categorical data were expressed as proportions and compared using the chi-squared test. The PSM method was used to reduce bias caused by potential confounding factors. A 1:1 PSM was performed using greedy nearest neighbor matching and the caliper was set as 0.01. Multivariate logistic regression analysis was then utilized to analyze the association between various factors and AKI risk. Meanwhile, Kaplan–Meier survival analysis was performed to assess the prognosis of patients with or without the use of DAPA. The Log-rank test was used to compare differences between the two groups. *P*-values less than 0.05 were considered statistically significant. Statistical analysis was conducted with SPSS software (IBM SPSS Statistics, version 25.0). Prism 8.0 (GraphPad, Inc.) was used for graphing.

## Results

### Patient characteristics

In total, 1839 patients diagnosed with AMI were included in the study. Among them, 1358 (73.8%) cases were male. The mean age of the enrolled patients was 64.6 ± 13.9 years. The baseline characteristics are shown in Table 1. There were 278 patients in the DAPA group and 1561 in the Ctrl group. Patients in the DAPA group were younger and had higher body mass index (BMI), diastolic blood pressure, heart rate, and poor cardiac function at the time of admission (*P* < 0.05).

**Table 1:** Baseline characteristics of patients in the entire cohort

	**Ctrl group**	**DAPA group**	***P*-value**
	**(*N* = 1561)**	**(*N* = 278)**	
**Demographic characteristics**			
Age, (y)	65.1 ± 14.0	62 ± 13.2	0.001
Male, *n* (%)	1146 (73.4%)	212 (76.3%)	0.32
BMI, kg/m2	24.2 ± 3.6	25.7 ± 3.8	<0.001
Systolic pressure, mmHg	130.7 ± 24.0	133.3 ± 24.1	0.091
Diastolic pressures, mmHg	78.4 ± 16.0	81.2 ± 14.7	0.007
Heart rate, bpm	80.7 ± 16.7	82.6 ± 14.5	0.046
Hypertension, *n* (%)	1035 (66.3%)	216 (77.7%)	<0.001
Diabetes mellitus, *n* (%)	402 (25.8%)	263 (94.6%)	<0.001
Smoking, *n* (%)	739 (47.3%)	120 (43.2%)	0.199
Drinking, *n* (%)	171 (11.0%)	34 (12.2%)	0.533
STEMI, *n* (%)	982 (62.9%)	171 (61.5%)	0.657
Killip ≥ 3, *n* (%)	184 (11.8%)	41 (14.8%)	0.165
**Biochemical examinations**			
White cell count, × 10^9/L	9.7 ± 3.6	10.4 ± 3.5	0.004
Hemoglobin, g/l	137.4 ± 21.0	145.8 ± 19.4	<0.001
Uric acid, µmol/l	355.5 ± 113.1	335.1 ± 108.4	0.005
Triglycerides, mmol/l	1.6 ± 1.2	2.3 ± 2.0	<0.001
Cholesterol, mmol/l	4.3 ± 2.0	4.4 ± 1.3	0.476
TSH, mIU/L	1.9 ± 4.2	1.6 ± 3.0	0.357
Baseline eGFR, ml/min/1.73m2	93.2 ± 41.7	116.6 ± 42.8	<0.001
Baseline creatinine, µmol/l	82.5 ± 34.9	73.0 ± 23.7	<0.001
Fasting glucose, mmol/l	8.61 (2.92)	8.90 (2.14)	0.109
NT-proBNP, pg/ml	678 (182, 2732.5)	757 (256.3, 2377.5)	0.622
LVEF, %	50.8 ± 9.2	49.5 ± 10.2	0.055
**Interventional therapy**			
Stent, *n* (%)	1304 (83.5%)	231 (83.1%)	0.855
LM, *n* (%)	14 (0.9%)	1 (0.4%)	0.579
LAD, *n* (%)	677 (43.4%)	135 (48.6%)	0.108
RCA, *n* (%)	446 (28.6%)	73 (26.3%)	0.43
LCX, *n* (%)	254 (16.3%)	50 (18.0%)	0.478
Contrast exposure time > 60 min, *n* (%)	318 (20.4%)	80 (28.8%)	0.002
**Medication**			
ACEI, *n* (%)	1010 (64.7%)	204 (73.4%)	0.005
β-blockers, *n* (%)	1020 (65.3%)	201 (72.3%)	0.024
**Postoperative outcomes**			
AKI, *n* (%)	314 (20.1%)	37 (13.3%)	0.008
Death, *n* (%)	90 (5.8%)	7 (2.5%)	0.026

Data are presented as mean ± SD for continuous variables and as proportions for categorical variables

*BMI* body mass index, *STEMI* ST-segment elevation myocardial infarction, *TSH* thyroid-stimulating hormone, *eGFR* estimated glomerular fltration rate, *NT-proBNP* N-terminal pro–brain natriuretic peptide, *LVEF* left ventricular ejection fraction, *LM* left main, *LAD* left anterior descending, *LCX* left circumflex, *RCA* right coronary artery, *ACEI* angiotensin-converting enzyme inhibitor, *AKI* acute kidney injury

### Independent predictors for AKI

**Fig. 2:**
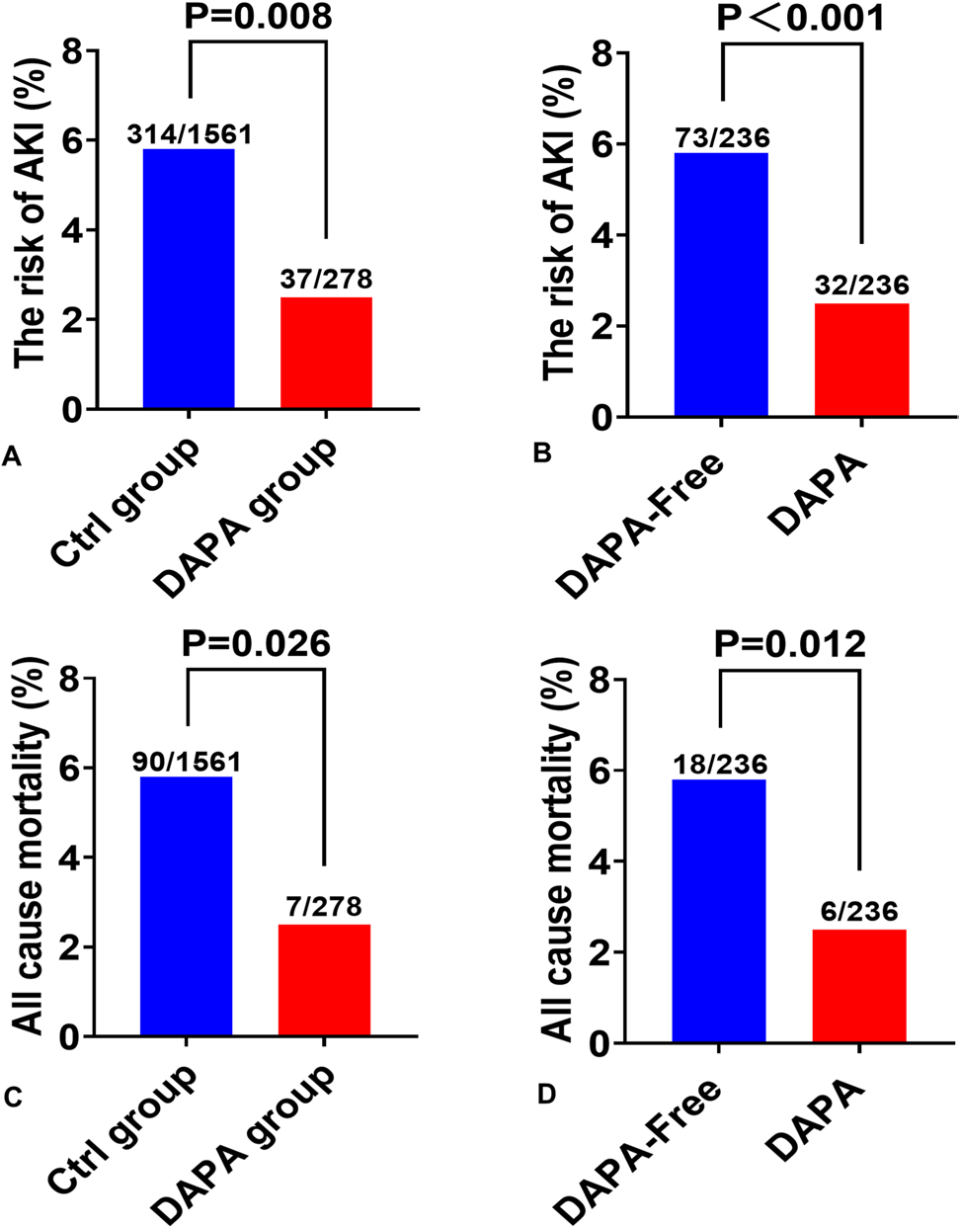
Proportion of AKI and all-cause mortality in patients with AMI. **A** The incidence of AKI between two groups in the entire cohort. **B** The incidence of AKI between two groups in the propensity-scored matched cohort. **C** The incidence of all-cause mortality between two groups in the entire cohort. **D** The incidence of all-cause mortality between two groups in the propensity-scored matched cohort

The incidence of AKI in the entire cohort was 351 (19.1%). The incidence of AKI was significantly higher in the Ctrl group than in the DAPA group (20.1% vs. 13.3%, *P* = 0.008) (Fig. 2A). Then we assessed the association of DAPA and the risk of postoperative AKI with logistic regression analysis. The univariate analysis showed that age, sex, BMI, heart rate, hypertension, smoking, Killip class, hemoglobin, DAPA, and eGFR were independent influence factors of postoperative AKI (Table 2). Moreover, the multivariate analysis showed that DAPA (OR 0.66; 95% CI 0.44–0.97; *P* = 0.036), BMI, heart rate, hypertension, Killip class, and hemoglobin were independent influence factors (Table 2). These results indicated that DAPA was associated with a reduced risk of postoperative AKI in AMI patients.

**Table 2:** Logistic regression analysis for risk factors associated with acute kidney injury in the entire cohort

**Variables**	**Univariable analysis**			**Multivariable analysis**		
	**OR**	**95% CI**	***P*-value**	**OR**	**95% CI**	***P*-value**
Dapagliflozin	0.61	0.42–0.88	0.008	0.66	0.44–0.97	0.036
Age	1.03	1.02–1.04	<0.001	1.01	0.99–1.02	0.415
Male	0.60	0.46–0.77	<0.001	0.9	0.64–1.27	0.539
BMI	0.92	0.89–0.95	<0.001	0.95	0.92–0.99	0.011
Systolic pressure	1.00	1.00–1.01	0.401	-	-	-
Diastolic pressures	1.00	0.99–1.01	0.953	-	-	-
Heart rate	1.02	1.01–1.03	<0.001	1.02	1.01–1.02	<0.001
Hypertension	1.79	1.36–2.35	<0.001	1.85	1.38–2.48	<0.001
Diabetes mellitus	1.20	0.94–1.52	0.136	-	-	-
Smoking	0.73	0.58–0.93	0.009	1.16	0.85–1.57	0.348
Drinking	0.70	0.47–1.05	0.087	-	-	-
STEMI	1.00	0.79–1.27	0.993	-	-	-
Killip ≥ 3	3.08	2.28–4.15	<0.001	2.27	1.63–3.17	<0.001
White cell count	1.01	0.98–1.05	0.431	-	-	-
Hemoglobin	0.98	0.97–0.98	<0.001	0.99	0.98–0.99	<0.001
Triglycerides	0.93	0.84–1.03	0.168	-	-	-
Cholesterol	0.95	0.86–1.06	0.366	-	-	-
TSH	0.99	0.95–1.02	0.456	-	-	-
Egfr	0.99	0.99–1.00	<0.001	1.00	1.00–1.00	0.589

*BMI* body mass index, *STEMI* ST-segment elevation myocardial infarction, *TSH* thyroid-stimulating hormone, *eGFR* estimated glomerular fltration rate

### Propensity matching analysis for AKI

The bias and confounding variables of the baseline data may affect the conclusions in observational studies. Therefore, we used the propensity matching analysis to reduce the influence of these biases and confounding variables. According to the baseline characteristics of the enrolled patients in Table 1, age, sex, BMI, eGFR, and diabetes mellitus were chosen as matching variables. Finally, 472 patients were matched, without significant differences in baseline characteristics between the two groups, except for hemoglobin and triglycerides levels (Supplementary Table 1). The proportion of patients with AKI was significantly lower in the DAPA group than in the DAPA-Free (30.9% vs. 13.6%, *P* < 0.001) (Fig. 2B).

The association of DAPA with the risk of AKI in the matched cohort was assessed. We found that DAPA reduced postoperative AKI risk by 65% in the univariable analysis (OR 0.35; 95% CI, 0.22–0.56; *P* < 0.001). After adjustment, DAPA still reduced the postoperative risk of AKI significantly in patients with AMI (OR 0.32; 95% CI, 0.19–0.53; *P* < 0.001) (Supplementary Table 2).

### Follow up

The follow-up in the entire cohort of this study lasted a median of 724 days (interquartile range, 715–732 days). The incidence of all-cause mortality was significantly higher in the Ctrl group than in the DAPA group (5.8% vs. 2.5%, *P* = 0.026) (Fig. 2C). In the matched cohort, the patients in the DAPA group also had a significantly lower all-cause mortality compared with the DAPA-free group (7.6% vs. 2.5%, *P* = 0.012) (Fig. 2D).

**Fig. 3:**
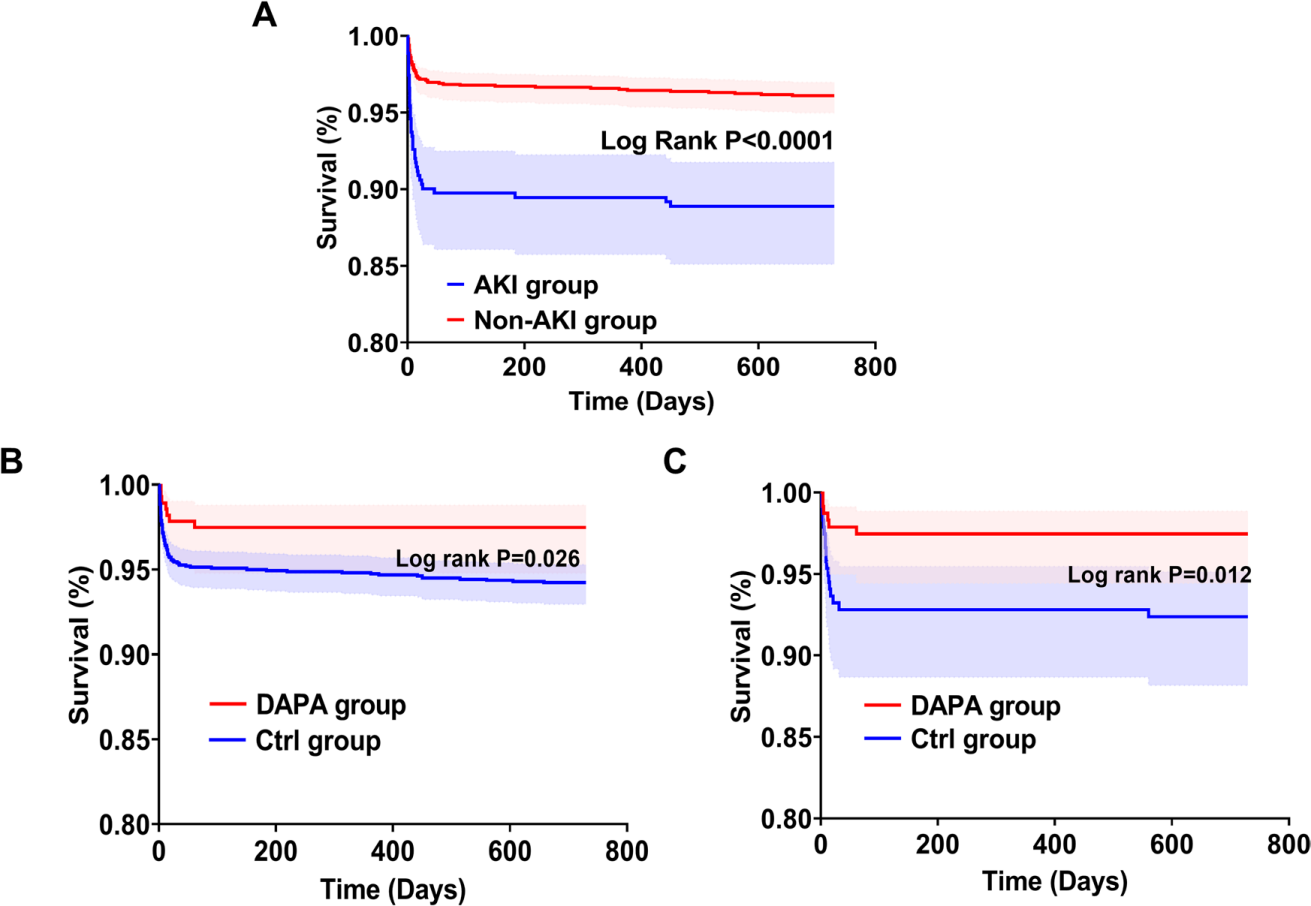
Survival analyses according to dapagliflozin treatment and the prevalence of AKI before and after matching. **A** Kaplan–Meier survival curve stratified by postoperative AKI in the entire cohort. **B** Association between dapagliflozin and mortality in the entire cohort. **C** Association between dapagliflozin and mortality in the matched cohort

Kaplan–Meier survival analysis showed that compared with the non-AKI group, the risk of all-cause mortality was significantly higher in the AKI group (log-rank *P* < 0.001) (Fig. 3A). Among all the patients, the risk of all-cause mortality was significantly reduced in the DAPA group than in the Ctrl group (log-rank *P* = 0.026) (Fig. 3B). Consistent with the results before matching, the patients in the DAPA group still presented with a lower risk of all-cause mortality after matching (log-rank *P* = 0.012) (Fig. 3C).

## Discussion

Ischemic heart disease and the resulting heart failure are the most important cause of death in the global population. AMI is a severe subtype of ischemic heart disease, with a high mortality and a poor prognosis. There are two main findings in this study. First, SGLT2 inhibitor DAPA was associated with a lower risk of postoperative AKI in AMI patients after PCI therapy. Second, it also significantly reduced all-cause mortality in AMI patients after PCI therapy.

The incidence of AKI in AMI hospitalized patients varies across studies, ranging from 10 to 30% [19–23]. In the current study, we found that the incidence of AKI was 19.1%. Here are possible reasons for this higher incidence. First, a higher proportion of patients in this study had comorbid underlying diseases, such as hypertension and diabetes mellitus. Second, there were more elderly patients in our study, and therefore baseline eGFR levels may be lower than in younger patients. Finally, different diagnostic criteria for AKI and the time of retesting serum creatinine might have also affected the incidence of AKI.

In the current study, we found that the use of SGLT2 inhibitor DAPA significantly reduced the risk of AKI in AMI patients. In clinical and experimental studies, DAPA was suggested to improve renal function. It is reported that DAPA may attenuate contrast-induced AKI by inhibiting HIF-1α/HE4/NF-κB signaling in vitro and in vivo [24]. Another study has shown that DAPA attenuates endotoxic shock associated with lipopolysaccharide-induced AKI [25], which may involve the activation of the AMPK pathway. It was also found that SGLT2 inhibitors could activate anti-inflammatory and antifibrotic pathways, improve renal oxygenation, and reduce glomerular hypertension and hyperfiltration, in addition to their obvious benefits in lowering blood pressure and reducing body weight [26, 27]. SGLT2 inhibitors have also been linked to reductions in oxidative stress, inflammatory markers (nuclear factor κB, interleukin 6, monocyte chemotactic protein-1, macrophage infiltration), and fibrosis (fibronectin, transforming growth factor-β) [27, 28]. DAPA osmotic diuresis leads to decreases in plasma volume and blood pressure, which activates a reflex known as tubulo-glomerular feedback, which in turn increases sodium reuptake by macular cells. This leads to vasoconstriction of afferent small arterioles and a decrease in intraglomerular pressure, which ultimately reduces RAAS-mediated activity [29]. Ultimately, long-term renal protection is achieved.

Several studies have shown that SGLT2 inhibitors significantly reduce cardiovascular mortality in DM patients with heart failure. The DAPA-HF [30] study has evaluated the long-term effects of SGLT2 inhibitor DAPA versus placebo on morbidity and mortality in patients with heart failure plus reduced ejection fraction (HFrEF). The results suggested that DAPA decreased the rate of the primary endpoint (compound endpoint of worsening heart failure or cardiovascular death) by 26%, significantly lowered the rates of both independent endpoints, and in addition, DAPA reduced the risk of all-cause mortality and improved heart failure symptoms, physical function, and quality of life. The EMPEROR-reduced [31] has suggested that empagliflozin significantly reduces the risk of cardiovascular death or hospitalization for heart failure by 25% in HFrEF patients. The study included patients with eGFR > 15 mL/min/1.73 m^2^, and the results showed that empagliflozin could delay the decline of eGFR in the enrolled patients. Clinical studies and experimental studies on SGLT2 inhibitor treatment after AMI were limited [32]. In the current study, we observed that DAPA treatment was significantly associated with a reduced risk of all-cause death in patients with AMI after PCI.

DAPA, a SGLT2 inhibitor, is widely applied as an oral hypoglycemic drug in clinical practice. SGLT-2 inhibitors can reduce sodium and glucose reabsorption in the proximal tubule, and thus increase urinary glucose and sodium excretion [33, 34]. SGLT2 inhibitors have shown impressive positive effects in patients with heart failure, but the molecular mechanisms behind its clinical benefits have not been intensely investigated. It has been reported that DAPA attenuates cardiac fibrosis through regulation of macrophage polarization via STAT3 signaling in infarcted rat hearts [35]. In a DM and non-DM rat AMI model, canagliflozin shrinks the infarct size of the injured heart. This study demonstrates that the infarct-sparing effect of canagliflozin results from a glucose-independent effect [36]. In the present study, we found that DAPA reduced all-cause mortality in AMI patients. The underlying mechanisms may be explained as follows: first, the natriuresis-related hemodynamic effects of SGLT2 inhibitors, including lowering blood pressure and diuresis, reduce the patient’s capacity load. Second, the use of DAPA lowers central systolic blood pressure and thus central pulse pressure, suggesting that DAPA treatment may reduce arterial stiffness [37].

There are some limitations of this study. First, this is a retrospective observational study conducted in a single center. The sample size of the present study was relatively small. Thus, in the future, multi-center prospective studies are still needed. Second, although no significant differences were observed in fasting glucose levels between the two groups, some information on glycemic control of the enrolled patients during hospitalization and follow-up time missed, making it impossible to carry out comparisons. Third, serum creatinine levels changed dynamically after PCI; we can only retest them at fixed time points. It is unclear whether the long-term application of SGLT2 inhibitors could improve long-term prognosis in AMI patients.

## Conclusions

SGLT2 inhibitor DAPA was associated with lower risks of incident AKI and all-cause mortality in AMI patients after PCI therapy.

### Supplementary Information

Below is the link to the electronic supplementary material.

Supplementary file1 (DOCX 22 KB)

## Data Availability

The datasets and materials used in the study are available from the corresponding author.
