# Supplementary material for: Association of SGLT2 inhibitor dapagliflozin with risks of acute kidney injury and all-cause mortality in acute myocardial infarction patients
Source: Eur J Clin Pharmacol. 2024 Feb 6;80(4):613–20. doi: 10.1007/s00228-024-03623-7 (PMC10937750; doi:10.1007/s00228-024-03623-7)
Supplement: Supplementary file 1 — Supplementary file1 (DOCX 22 KB) [file 228_2024_3623_MOESM1_ESM.docx]

**Supplementary table 1.** Baseline characteristics of patients in the propensity scored matched cohort.

|  | **Ctrl group**  **(N = 236)** | **DAPA group**  **(N = 236)** | ***P*-value** |
| --- | --- | --- | --- |
|  |  |  |  |
| **Demographic characteristics** | | | |
| Age, (y) | 63.2±12.2 | 62.9±13.0 | 0.778 |
| Male, n (%) | 184(78.0%) | 175(74.2%) | 0.332 |
| BMI, kg/m2 | 25.3±3.7 | 25.3±3.4 | 0.916 |
| Systolic pressure, mmHg | 133.0±23.9 | 133.2±24.2 | 0.928 |
| Diastolic pressures, mmHg | 80.8±17.0 | 80.8±14.8 | 0.988 |
| Heart rate, bpm | 82.6±17.7 | 82.2±14.4 | 0.818 |
| Hypertension, n (%) | 182(77.1%) | 183(77.5%) | 0.912 |
| Diabetes mellitus, n (%) | 221(93.6%) | 221(93.6%) | 1.000 |
| Smoking, n (%) | 111(47.0%) | 101(42.8%) | 0.355 |
| Drinking, n (%) | 26(11.0%) | 32(13.6%) | 0.400 |
| STEMI, n (%) | 146(61.9%) | 145(61.4%) | 0.925 |
| Killip ≥3, n (%) | 40(17.0%) | 35(14.8%) | 0.529 |
| **Biochemical examinations** | | | |
| White cell count, ×10^9/L | 10.0±4.0 | 10.4±3.5 | 0.344 |
| Hemoglobin, g/l | 140.2±18.6 | 144.±20.1 | 0.013 |
| Uric acid, µmol/l | 336.4±106.4 | 334.4±106.9 | 0.832 |
| Triglycerides, mmol/l | 1.8±1.5 | 2.2±1.9 | 0.019 |
| Cholesterol, mmol/l | 4.3±1.1 | 4.4±1.3 | 0.305 |
| TSH, mIU/L | 1.9±4.7 | 1.7±3.2 | 0.595 |
| Baseline eGFR, ml/min/1.73m2 | 109.0±53.0 | 112.9±41.0 | 0.362 |
| Baseline creatinine, µmol/l | 74.5±34.0 | 74.7±24.6 | 0.931 |
| NT-proBNP, pg/ml | 561(156,2443) | 770(277,2352.5) | 0.190 |
| LVEF, % | 50.9±9.1 | 49.4±10.2 | 0.090 |
| **Interventional therapy** |  |  |  |
| Stent, n (%) | 200(84.8%) | 195(82.6%) | 0.533 |
| LM, n (%) | 5(2.1%) | 1(0.4%) | 0.218 |
| LAD, n (%) | 99(42.0%) | 115(48.7%) | 0.139 |
| RCA, n (%) | 71(30.1%) | 62(26.3%) | 0.357 |
| LCX, n (%) | 40(17.0%) | 40(17.0%) | 1.000 |
| Contrast exposure time > 60min, n (%) | 50(21.1%) | 57(24.2%) | 0.442 |
| **Medication** |  |  |  |
| ACEI, n (%) | 157(66.5%) | 170(72.0%) | 0.195 |
| β-blockers, n (%) | 155(65.7%) | 167(70.8%) | 0.236 |
| **Postoperative outcomes** | | | |
| AKI, n (%) | 73(30.9%) | 32(13.6%) | <0.001 |
| Death, n (%) | 18(7.6%) | 6(2.5%) | 0.012 |

Data are presented as mean ± SD for continuous variables and as proportions for categorical variables. BMI, body mass index; STEMI, ST-segment elevation myocardial infarction; TSH, thyroid-stimulating hormone; eGFR, estimated glomerular fltration rate; NT-proBNP, N-terminal pro–brain natriuretic peptide; LVEF, left ventricular ejection fraction; LM, left main; LAD, left anterior descending; LCX, left circumflex; RCA, right coronary artery; ACEI, angiotensin-converting enzyme inhibitor; AKI, acute kidney injury.

**Supplementary table 2.** Logistic regression analysis for risk factors associated with acute kidney injury in the propensity scored matched cohort.

| **variables** | **Univariable analysis** | | | **Multivariable analysis** | | |
| --- | --- | --- | --- | --- | --- | --- |
|  | **OR** | **95% CI** | ***P*-value** | **OR** | **95% CI** | ***P*-value** |
| Dapagliflozin | 0.35 | 0.22-0.56 | <0.001 | 0.32 | 0.19-0.53 | <0.001 |
| Age | 1.02 | 1.00-1.04 | 0.019 | 1.00 | 0.97-1.02 | 0.663 |
| Sex | 0.48 | 0.30-0.77 | 0.002 | 0.51 | 0.29-0.88 | 0.016 |
| BMI | 0.89 | 0.83-0.95 | <0.001 | 0.89 | 0.83-0.96 | 0.002 |
| Heart rate | 1.02 | 1.01-1.03 | 0.005 | 1.02 | 1.00-1.03 | 0.053 |
| Hypertension | 2.71 | 1.42-5.16 | 0.002 | 3.29 | 1.62-6.68 | 0.001 |
| Killip ≥ 3 | 3.30 | 1.96-5.57 | <0.001 | 2.33 | 1.25-4.36 | 0.008 |
| Hemoglobin | 0.98 | 0.97-0.99 | <0.001 | 0.99 | 0.98-1.01 | 0.413 |

BMI, body mass index.
